# Regulating *Shaker* Kv channel clustering by hetero-oligomerization

**DOI:** 10.3389/fmolb.2022.1050942

**Published:** 2023-01-09

**Authors:** Esraa Nsasra, Guy Peretz, Irit Orr, Ofer Yifrach

**Affiliations:** Department of Life Sciences and the School of Brain Sciences and Cognition, Ben-Gurion University of the Negev, Beersheba, Israel

**Keywords:** hetero-oligomerization, scaffold proteins, alternative splicing, potassium chanels, clustering, action potential, subunit assembly

## Abstract

Scaffold protein-mediated voltage-dependent ion channel clustering at unique membrane sites, such as nodes of Ranvier or the post-synaptic density plays an important role in determining action potential properties and information coding. Yet, the mechanism(s) by which scaffold protein-ion channel interactions lead to channel clustering and how cluster ion channel density is regulated are mostly unknown. This molecular-cellular gap in understanding channel clustering can be bridged in the case of the prototypical *Shaker* voltage-activated potassium channel (Kv), as the mechanism underlying the interaction of this channel with its PSD-95 scaffold protein partner is known. According to this mechanism, changes in the length of the intrinsically disordered channel C-terminal chain, brought about by alternative splicing to yield the short *A* and long *B* chain subunit variants, dictate affinity to PSD-95 and further controls cluster homo-tetrameric Kv channel density. These results raise the hypothesis that heteromeric subunit assembly serves as a means to regulate Kv channel clustering. Since both clustering variants are expressed in similar fly tissues, it is reasonable to assume that hetero-tetrameric channels carrying different numbers of high- (*A*) and low-affinity (*B*) subunits could assemble, thereby giving rise to distinct cluster Kv channel densities. Here, we tested this hypothesis using high-resolution microscopy, combined with quantitative clustering analysis. Our results reveal that the *A* and *B* clustering variants can indeed assemble to form heteromeric channels and that controlling the number of the high-affinity *A* subunits within the hetero-oligomer modulates cluster Kv channel density. The implications of these findings for electrical signaling are discussed.

## Introduction

Action potential (AP) generation and propagation and the evoked synaptic potential all rely on precisely timed events associated with gating transitions of voltage-dependent Na^+^ and K^+^ channels (Kandel et al., 2000) clustered in multiple copies at unique membrane sites, such as the initial segment of an axon, nodes of Ranvier or the post-synaptic density (PSD) (Lai & Jan, 2006; Trimmer, 2015). While it is clear that changes in ionic current shape, reflecting changes in the temporal dimension, affect action potential conduction properties (Debanne et al., 1997; Giese et al., 1998; Johnston et al., 1999), only recently have we begun to realize the importance of how changes in the density of voltage-dependent ion channels at their targeted sites of expression, reflecting changes in the spatial dimension, affect AP properties and information coding (Århem, et al., 2006; Zeberg et al., 2010; Zeberg et al., 2015; Freeman et al., 2016; Pfeiffer et al., 2020). Despite emerging evidence attesting to the importance of ion channel density for efficient electrical signaling and information coding, little is currently known of the process of voltage-dependent ion channel clustering or its regulation (Nirenberg & Yifrach, 2020). It is clear that voltage-dependent ion channel clustering is an active process, involving channel interactions with specific members of one of several scaffold protein families (Good et al., 2011). For example, Nav channel clustering at nodes of Ranvier is mediated by interaction of the channel with the ankyrin G scaffold protein (Zhou et al., 1998; Komada & Soriano, 2002), while Kv channel clustering at the PSD of excitatory synapses is mediated by binding to the PSD-95 scaffold protein (Kim et al., 1995; Tejedor et al., 1997; Tiffany et al., 2000; Ruiz-Canada et al., 2002). In both cases, however, the mechanism by which elementary binding events lead to the clustering of many ion channel molecules in a restricted membrane area and the regulation of this process remain largely unclear. In the absence of a detailed molecular mechanism describing ion channel-scaffold protein interactions, bridging this molecular-cellular gap to understand voltage-gated ion channel clustering has proven challenging.

**FIGURE 1:**
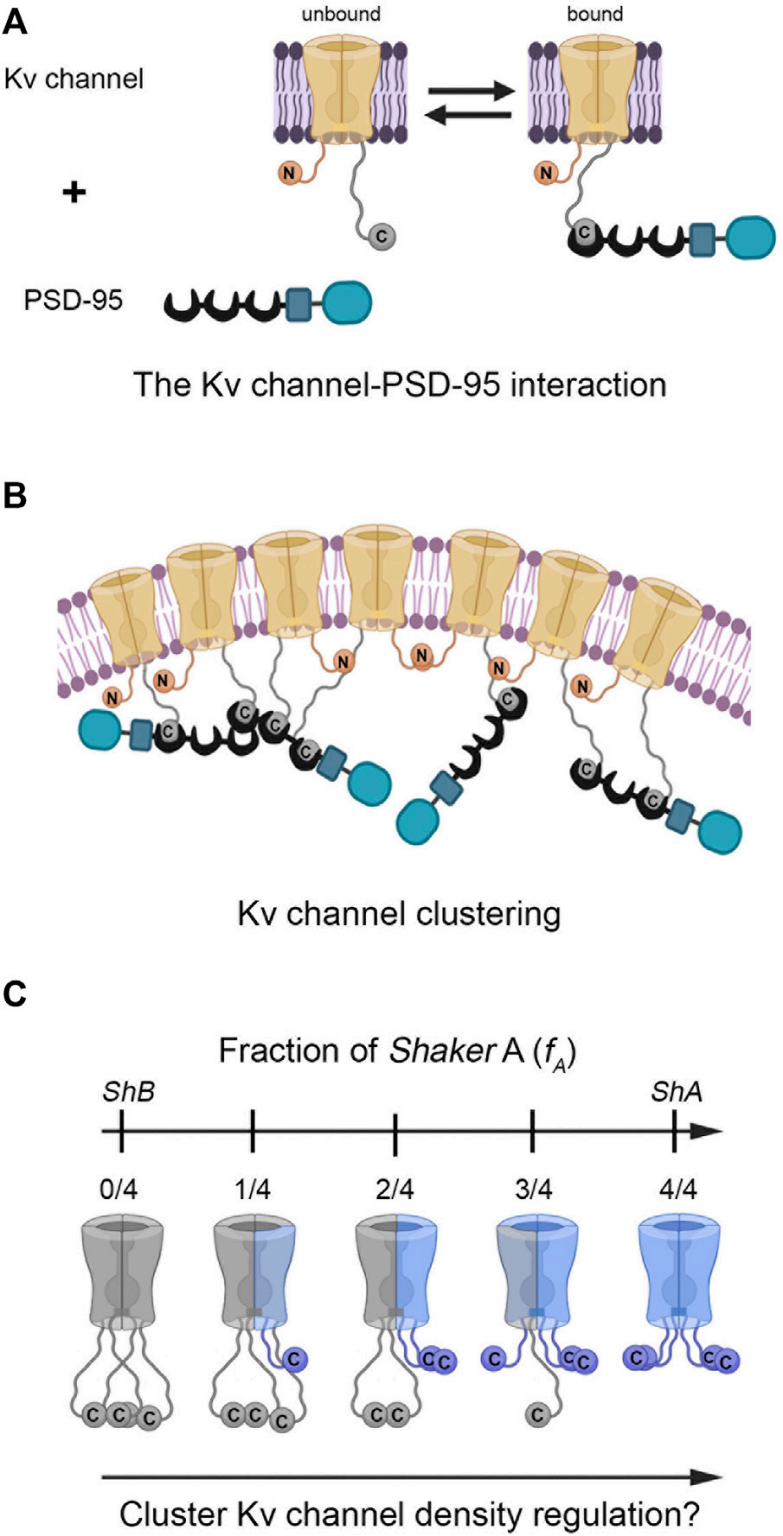
Does hetero-oligomeric Kv channel subunit assembly regulate PSD-95-mediated Kv channel clustering? **(A)** Schematic representation of the ‘ball and chain’-like mechanism for Kv channel binding to the PSD-95 scaffold protein. In this mechanism, the extended 'chain' at the channel C-terminal binds PSD-95 upon interaction of the chain-tethered peptide ‘ball’ with PSD-95 PDZ domain(s) in a manner reminiscent of the process of fast (N-type) channel inactivation. The Kv channel-PSD-95 interaction is thus entropy-controlled, dictated by Kv channel 'chain' length. Given the stoichiometry of the interaction (Gomperts, 1996) and the ability of PSD-95 to aggregate (Hsueh et al., 1997), channel clustering occurs **(B)**. Note that clustering model is only an illustrative schematic model, the exact details of which await experimental verification and which are beyond the scope of the current study. The membrane-embedded portion of the Kv channel corresponds to the voltage-sensor and pore domains. The crescent, rectangular and rounded box shapes represent the PDZ, SH3 and guanylate kinase-like domains of the PSD-95 protein, respectively. For clarity, only one pair of N- and C-terminal chains of the tetramers are shown in panels A and B. **(C)** Heterologous subunit assembly of both *Shaker A* (light blue) and *B* (gray) variants can produce tetrameric channels with different numbers of the high-affinity *A* subunits, potentially giving rise to distinct PSD-95-Kv channel affinities and cluster Kv channel densities. For clarity, the intrinsically-disordered N-terminal inactivation chains were omitted from the channel drawings.

The prototypical *Shaker* Kv channel protein offers an excellent model system to address the questions raised above since the mechanism underlying its interaction with the PSD-95 scaffold protein has been elucidated (Magidovich et al., 2007; Zandany et al., 2015). In this interaction, the random walk motion of the unstructured C-terminal channel ‘chain’, bearing a conserved PDZ-binding sequence motif (the ‘ball’) at its tip, recruits the PSD-95 scaffold protein partner (Figure 1A) (Zandany et al., 2015). This mechanism is reminiscent of the role of the N-terminal tail in regulating channel fast inactivation, as described by the ‘ball and chain’ mechanism (Hoshi et al., 1990; Zandany et al., 2015). Evidence supporting the proposed mechanism of Kv channel-PSD-95 interaction relying on the ‘chain’-length dependence of thermodynamic and kinetic parameters controlling the interaction (Zandany et al., 2015; Lewin et al., 2019), as predicted by polymer chain theory (Timpe and Peller, 1995), has been provided. For instance, both the affinity and entropy of the binding reaction were found to linearly depend on Kv channel ‘chain’ length (Zandany et al., 2015; Lewin et al., 2019). Furthermore, the rate constant of association between both proteins revealed an expected power law dependence on 'chain' length (Lewin et al., 2019). Based on these findings, it has been argued that the Kv channel C-terminal sequence functions as an entropic clock (Dunker et al., 2001; Uversky & Dunker, 2010) that times PSD-95 binding, with ‘chain’ length acting as the hands of the clock (Zandany et al., 2015; Lewin et al., 2019). Indeed, alternative splicing of the *Shaker* channel gene only involves the N- or C-terminal ‘chains’, yielding natural channel variants presenting different ‘chain’ lengths (Pongs et al., 1988; Schwarz et al., 1988). These 'chain' variants give rise to distinct kinetics of inactivation (Hoshi et al., 1990; Zagotta et al., 1990) and PSD-95 binding affinity (Zandany et al., 2015; Lewin et al., 2019), respectively.

The ‘ball and chain’-like mechanism that describes the Kv channel-PSD-95 interaction is molecular in essence, and as such, reveals no information on Kv channel clustering. One can thus ask what are the cellular correlates of the ‘ball and chain’-like mechanism for channel-scaffold protein binding, if these indeed exist (Figure 1B). Recently, we put forward an experimental paradigm aimed at understanding PSD-95-mediated Kv channel clustering (Lewin et al., 2020). Our strategy was based on a reliable heterologous expression system that supports Kv channel clustering and combines high-resolution confocal microscopy (Huff et al., 2017) with clustering analysis to provide a quantitative assessment of various channel clustering attributes, such as cluster area size, number of clusters per cell and most importantly, cluster ion channel density (Lewin et al., 2020). Using this setup, we showed that the homo-tetrameric Kv channel comprising the high-affinity short 'chain' (*A*) splice variant-generated subunit exhibited higher cluster Kv channel density than did the homomeric channel comprising low-affinity long-'chain' (*B*) subunits alone. Furthermore, examining the clustering metrics of a series of artificial Kv channel deletion variants presenting different C-terminal ‘chain’ lengths revealed a unique length-dependence of cluster Kv channel density that correlated with variant affinity to the PSD-95 scaffold protein partner (Lewin et al., 2020). Thus, Kv channel clustering reveals an entropy-based mode of regulation that mirrors the thermodynamic entropy signature of the Kv channel-PSD-95 molecular binding reaction described above (Zandany et al., 2015; Lewin et al., 2019).

The results showing that the native *A* and *B* homo-tetrameric channels present different C-terminal ‘chain’ lengths, affinities to scaffold proteins and cluster Kv channel densities, raise the hypothesis that heteromeric subunit assembly offers a means to regulate Kv channel clustering, in a manner analogous to the assembly of different inactivation splice variant-generated subunits (McCormack et al., 1990). Since both the *A* and *B* channel clustering variants exhibit an identical T1 assembly domain and are both expressed in similar fly tissues and during similar developmental stages (Mottes & Iverson, 1995; Rogero et al., 1997), it is only reasonable to assume that hetero-tetrameric channels carrying different numbers of high- (*A*) and low-affinity (*B*) subunits could assemble, thereby giving rise to distinct cluster Kv channel densities (Figure 1C). We tested this hypothesis using our reductionist clustering experimental design at the biochemical and cellular levels. This design provides a reliable and sensitive quantitative handle to assess ion channel clustering using cluster channel density metrics (Lewin et al., 2020). Our results provide clear cut evidence that Kv channel density within clustering sites is regulated by channel hetero-oligomerization.

## Materials and Methods

### Molecular biology, cell culture, transfection and immunostaining

For protein clustering analysis, both the PSD-95 and *Shaker* Kv channel proteins used in the current study were *Drosophila* proteins, with the S97 PSD-95 homologue being used. For a detailed description of the molecular biology, cell culture, transfection and immunostaining techniques used, refer to Lewin et al. (2020). To examine hetero-oligomeric Kv channel assembly *in vivo*, a bimolecular fluorescence complementation (BiFC) assay was employed (see below). For this, the 239 amino acid-long green fluorescent protein (GFP) was used as the target signaling protein. The DNA sequence encoding the first 154 N-terminal residues of GFP (N_GFP_) was amplified by standard PCR using the *Phusion* DNA polymerase enzyme. Likewise, the DNA sequence encoding the remaining C-terminal 85 amino acids of GFP (C_GFP_) was similarly amplified. To clone the respective GFP segments into the appropriate positions in the *Shaker A* and *B* Kv channel variants, one-step mutagenesis was performed. Briefly, first Kod Hot start DNA Polymerase was used to insert *SacII* restriction sites at DNA positions 76 and 509 of the respective *Shaker A* and *Shaker B* variants. Both insertion regions are spatially close, based on cryo-electron microscopy analysis of the *Shaker* Kv channel, thus enabling assembly of both folded GFP segments (Sokolova et al., 2001). Next, the N_GFP_ and C_GFP_ segments were fused to the N- and C-terminal domains of the respective pcDNA *ShA* and *ShB* variants using the engineered *SacII* sites and a Gibson reaction. Constructs were verified by colony PCR, *SacII* restriction reactions, and DNA sequencing. Plasmid constructs used are depicted in Supplementary Figure S1.

### Native membrane protein extraction

For protein analysis purposes, five plates of transfected SH-SY5Y cells reaching 90–95% confluence were used. Cells expressing the protein of interest were harvested upon medium removal and washing of adherent cells once with phosphate-buffered saline (PBS). Next, the cells were gently detached from the plate using a cell scraper, collected into a sterile tube, and washed thrice with PBS. Extraction of SH-SY5Y native and introduced membrane proteins was conducted using a standard protocol, essentially as described by Jett et al. (1997), with some modifications. Briefly, to permeabilize cell membranes, sedimented cells were re-suspended in S-buffer (50 mM Hepes, 0.3 M NaCl, 2 mM EDTA, 2 mM DTT and protease inhibitor cocktail) with 1.2% L-dodecyldimethylaminoxide (LDAO) for 30 min on ice (4°C). Following ultracentrifugation for 1 h at 100,000 g, the supernatant containing detergent-soluble membrane proteins was collected and loaded onto the appropriate affinity column (see pull-down analysis, below).

### Pull-down analysis

A batch mode pull-down experimental setup was used to probe for hetero-oligomeric Kv channel binding using affinity columns to capture the channel either *via* the *A* or *B* subunit variants. Briefly, the total native membrane protein fraction (T) of SH-SY5Y cells transfected to express both His_6_-tagged *Shaker A* and FLAG-tagged *Shaker B* subunit variants was extracted (see above) and applied to either a His Trap or anti-*B* antibody affinity column. Before sample loading, the Ni^2+^ bead-containing column was washed with Ni-buffer (0.2 M NaCl, 50 mM Tris-HCl, pH 8, 10 mM imidazole, 10% glycerol and 0.5 mM EDTA), while the anti-FLAG column was washed with α-FLAG buffer (25 mM Hepes, pH 7.5, 50 mM NaCl, 5% glycerol, 0.5 mM DTT and 0.5 mM EDTA). Next, identical membrane protein extraction fractions were loaded onto the Ni^2^ and anti-FLAG columns and incubated for 1 h at 4°C with rotation. The flowthrough (FT) fractions were collected, as were the subsequent wash (W) fractions using the appropriate loading buffer. Elution of the bound sample (E) was achieved by applying free imidazole or the FLAG ligand, as appropriate. Proteins in the elution fractions of both columns were precipitated with 10% TCA, washed with acetone, and resuspended in SDS-PAGE sample buffer containing protein inhibitor cocktail. All fractions were then subjected to SDS-PAGE and Western blotting analyses using either anti-His (*A*) or anti-FLAG (*B*) antibodies.

### Split GFP bio-complementation assay

A GFP bio-complementation assay was performed as described by Kerppola, (2008). Briefly, DNA encoding for the structurally complementing N- or C-terminal halves of GFP (N_GFP_ and C_GFP_) were inserted into either the *A* or *B* subunit-encoding DNA at different sites known to be in close spatial proximity in the protein, based on the low-resolution electron microscopy structure of the native *Shaker K*v channel (Sokolova et al., 2001). N_GFP_ was inserted following position 76 of *Shaker A*, whereas C_GFP_ was inserted following amino acid 509 of *Shaker B*. DNA encoding for the both *Shaker A*-N_GFP_ and *Shaker B*-C_GFP_ fusion proteins were then transfected into SH-SY5Y cells. Following protein subunit expression, the cells were imaged using high-resolution confocal microscopy to detect any green fluorescence signal.

### Confocal microscopy surface expression and clustering analyses

To assess cellular Kv channel expression and clustering, the basal membrane area of fixed SH-SY5Y neuroblastoma cells transfected to express different concentration ratios of the *A* and *B* ‘chain’-length channel variants, with or without the PSD-95-GFP scaffold protein, was imaged using a Zeiss LSM880 inverted laser-scanning confocal microscope (Jena, Germany) equipped with an Airyscan high-resolution detection unit (Huff, 2015) under identical acquisition conditions. A Plain-Apochromat 63x/1.4 Oil DIC M27 objective was used, and parameters were set to avoid pixel intensity saturation and ensure Nyquist sampling in the XY plane (∼150 nm resolution). In our measurements, the actual axial resolution of the microscope was ∼350 nm (Huff, et al., 2017). Detection of FLAG-tagged Kv channel and PSD-95-GFP fusion proteins was achieved by focusing directly on the cover slip-attached basal membrane plane and measuring the red and green fluorescence signals using a 561 nm DPSS laser with a BP 570–620 emission filter and a 488 nm argon laser with a BP 495–550 emission filter, respectively. Under these conditions, the observed signals report on what is found on the plasma membrane, as explained in detail in our previous study (Lewin et al., 2020). To reduce experimental errors and allow accurate comparisons, care was taken to obtain cell images from samples subjected to the same transfection procedure and using identical acquisition conditions.

### Channel clustering metrics

Quantitative assessment of channel expression and clustering of the Kv channel subunit variants and combinations thereof was performed as previously described (Lewin et al., 2020). Briefly, the normalized channel surface expression level, the fraction of channels targeted to clusters, the number of channel clusters per cell, the mean cluster area size and cluster Kv channel density were evaluated and compared across transfection ratios. For calculating normalized channel surface expression, the channel-associated red fluorescence signal along each imaged cell area (*n* = ∼23) was integrated and the total fluorescence signal (*IFS*_*T*_) (reflecting channels residing within or between clusters) was divided by the cell area to yield the normalized PSD-95-mediated channel cell expression level. The other four PSD-95-mediated clustering attributes of the different Kv channel variants were quantitatively assessed using a two-step clustering metrics methodology. Briefly, Kv channel clustering sites within each co-transfected cell image (detected using the channel-associated red signal) were identified and counted using the 'Spot Counter' plug-in (https://github.com/nicost/spotCounter/) of the ImageJ analysis program (Schindelin et al., 2012). This plug-in detects local fluorescence maxima by scanning the image with a box of pre-defined size. Local maxima are accepted when the maximum is higher than a user-defined number, over the average of the four corners of the box. Images in which cluster spots were identified were then assessed using the 'Threshold-based segmentation' ImageJ macro-code that automatically identifies clustering sites, defines their borders and calculates cluster area and integrated florescence intensity within each cluster (*IFSc*, corrected for the image background signal). For each of the different Kv channel transfection ratio treatments, ∼23 cells were analyzed and a total of 3,000–5,000 clusters were evaluated for channel scaffold protein co-localization and subsequently measured to determine cluster area size and the integrated fluorescent signal. Cluster ion channel density was then evaluated by assessing fluorescent signal intensity obtained by dividing both quantities. The normalized cluster number per cell was calculated by dividing the number of channel clusters by cell area. The fraction of channels in clustering sites was calculated by integrating the channel-associated red fluorescence signal of all clusters (*IFSc*) and dividing by the total red fluorescence signal of the same cell (*IFS*_*T*_). For all cell expression and clustering parameters evaluated here, mean values and standard errors were obtained and are reported in Table 1. The code for the automated clustering analysis is freely available and can be found at the GitHub repository under the following address: https://github.com/esraan/MasterDegreeEsraa.git.

**TABLE 1:** The role of subunit hetero-oligomerization on PSD-95-mediated *Shaker* Kv channel clustering^a^.

Fraction of Shaker A [DNA] transfected^b^	Mean channel membrane surface expression ^c^ (IFS_T_/μm^2^) x10^4^	Mean number of clusters per cell^d^ (1/μm^2^) x10^−2^	Fraction of channels in clusters^e^	Mean cluster area size (μm^2^) x10^−2^	Mean cluster Kv channel density^f^ (IFSc/μm^2^) x10^4^
0.00	5.16 ± 0.21	22.48 ± 1.22	0.118 ± 0.007	1.49 ± 0.019	16.51 ± 0.10
0.25	4.90 ± 0.23	24.33 ± 1.75	0.144 ± 0.010	1.52 ± 0.018	17.89 ± 0.08
0.50	4.40 ± 0.23	25.96 ± 1.71	0.196 ± 0.009	1.61 ± 0.018	18.81 ± 0.09
0.75	5.74 ± 0.59	29.65 ± 2.42	0.190 ± 0.008	1.53 ± 0.015	20.62 ± 0.09
1.00	4.33 ± 0.25	27.80 ± 2.22	0.216 ± 0.014	1.56 ± 0.018	20.33 ± 0.09

^**a**^ Hetero-oligomerization effects on channel clustering were measured upon co-transfection of neuroblastoma SY5Y cells with different *A*:*B* subunit concentration ratios. Cell imaging was performed by high-resolution confocal microscopy combined with clustering analysis, as described in the text. Values and standard errors are reported for each variable.

^**b**^ The *A*:*B* subunit DNA concentration ratio co-transfected into cells.

^**c**^ Evaluated by integrating the total fluorescence red channel-associated cell signal (IFS_T_), normalized to cell area.

^**d**^ Obtained by counting the number of clusters per cell and normalized to cell area.

^e^ Obtained by dividing the IFS signal within clusters (IFSc) to the total cell IFS (IFS_T_).

^**f**^ Obtained by dividing the IFSc by cell area.

### Statistical analysis

To assess differences in clustering attributes among the different *A*/*B* transfection ratios using parametric ANOVA tests, normality and homo-scedasticity of the distributions obtained were first verified using Kolmogorov-Smirnov and Levene tests, respectively (Zar, 2010). Violation of both normality and homo-scedasticity assumptions led to data transformation. In cases where either normality or homo-scedasticity was the assumption violated, Welch or Kruskal–Wallis statistical tests were respectively performed (Zar, 2010). Among all clustering attributes tested, only the fraction of channels in clusters and the number of clusters per cell were found to be normally distributed and exhibited similar variance. Channel cell surface expression levels, on the other hand, only violated the normality assumption. The distributions of both cluster area size and cluster channel density violated both normality and homo-scedasticity assumptions, such that the data were transformed using logarithmic and square root transformations, respectively. An ANOVA test (or the non-parametric Welch test in the case of channel cell surface expression level metrics) was used to examine the *null* hypothesis that for any of the different channel metrics attributes, the values of the particular parameter (obtained for the different transfection ratios) were all the same. Rejection of the *null* hypothesis was based on a *p*-value smaller than 0.01. Following null hypothesis rejection for any particular clustering attribute addressed, Tuckey pairwise multiple comparisons (*post hoc*) were conducted to compare differences among the different transfection ratios. To test whether cluster channel density was correlated with *A/B* subunit transfection ratio, linear regression analysis was performed.

## Results

### Clustering variants assemble to form hetero-oligomeric Kv channels

**FIGURE 2:**
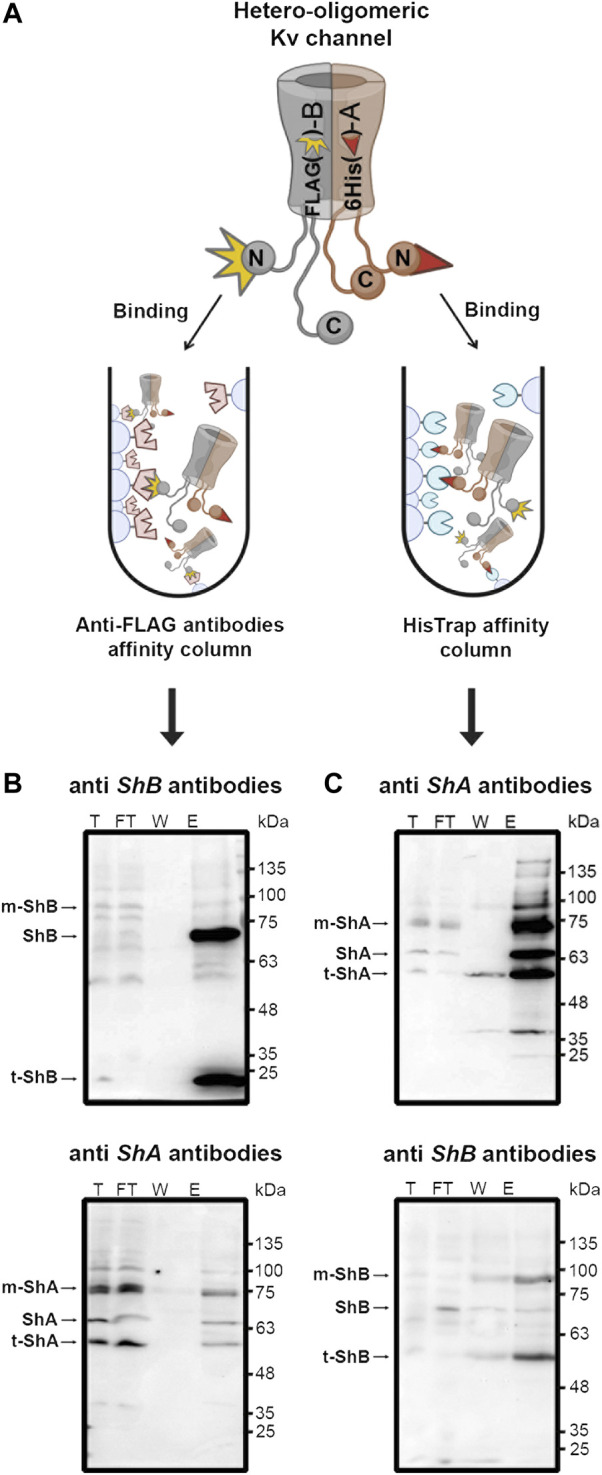
Pull-down analysis revealed hetero-oligomeric Kv channel molecules composed of both *A* and *B* subunit variants. **(A)** Schematic representation of the pull-down experimental setup used to detect hetero-oligomeric Kv channel subunit assembly (see text). Briefly, the native membrane protein fraction of SH-SY5Y cells expressing a 1:1 ratio of both the *A* and *B* Kv channel variants was loaded onto an affinity column presenting antibodies against the B variant or to a His-Trap (anti-A) affinity column. Following binding and washing, bound channels were eluted using the appropriate free ligand. The total (T), flow-through (FT), wash (W) and elution (E) fractions of the two affinity setups were subjected to SDS-PAGE and Western blot analyses involving antibodies against either the *A* or *B* channel variants **(B**,**C)**. **(B)** Western blot analyses of the eluant of a pull-down setup designed to capture the hetero-oligomeric Kv channel through the FLAG-tagged *B* subunit variant and probed with anti-FLAG and anti-His antibodies (upper and lower panels, respectively). The arrows indicate the bands corresponding to either the *Shaker A* or *B* channel variants present in the elution fractions. Bands corresponding to the lower molecular weight protein species were all found to be truncated *Shaker A* or *B* channel versions, as revealed by mass spectrometry. **(C)** Western blot analyses of the eluant of a pull-down setup designed to capture the hetero-oligomeric Kv channel through the His_6_ *-*tagged *A* subunit variant and probed with both anti-FLAG and anti-His antibodies (upper and lower panels, respectively). The '*m*' and '*t*' labels in panels **b** and **c** respectively refer to the mature (glycosylated) and truncated versions of the *A* and *B* variants, as verified by mass The experiments described in panels B and C were each repeated three times.

Hetero-oligomeric assembly of variant *Shaker* Kv channel subunits to generate fast inactivation functional diversity has been previously demonstrated using mRNA transcript co-injection into *Xenopus oocytes* followed by electrophysiological recordings (McCormack et al., 1990). To directly examine whether hetero-oligomeric assembly of the clustering *A* and *B* subunit variants to form hybrid Kv channel molecules is possible, we performed pull-down experiments involving the solvated native membrane protein fraction of SH-SY5Y cells transfected to express a 1:1 ratio of His_6_-tagged *A* and FLAG-tagged *B* channel subunit variants. Specifically, native membrane proteins were extracted from SH-SY5Y cells using a standard protocol (see Materials and Methods) and applied to either an anti-*A* His-trap affinity column or to an anti-*B* (anti-FLAG) antibody affinity column, thus allowing binding of the hybrid oligomeric channel particle *via* the appropriate tagged channel subunit (Figure 2A). Following washing and elution using the appropriate free imidazole or FLAG ligands, SDS-PAGE and Western blot analyses were performed. These analyses probed for the presence of the co-captured subunit variant using appropriate antibodies, with binding of both antibodies indicating the presence of hetero-oligomeric Kv channel particles comprising both *A* and *B* subunits. No antibody signal was detected in control experiments where the native membrane protein fraction of naïve cells (i.e., cells transfecting no *Shaker* channel proteins) was analyzed (not shown). Figure 2B delineates the results of a Western blot analysis of the total native membrane proteins (T), unbound flow-through (FT), wash (W) and elution (E) fractions of a pull-down experiment involving cells expressing tagged *Shaker* channel proteins using an anti-FLAG tag antibody affinity column to immobilize the Kv channel particle *via* the FLAG-tagged *B* subunit and probed using either anti-FLAG (upper panel) or anti-His antibodies (lower panel). The results described in the upper panel verify *B* subunit-based Kv channel immobilization, as reflected by the strong ∼70 kDa band in fraction E, corresponding to the long-chain tagged *Shaker B* subunit or its ∼25 kDa truncated version, as verified by mass spectrometry. The faint ∼100 kD band corresponds to the mature glycosylated species of the *B* channel (Papazian et al., 1995). Probing the same fractions using anti-His antibodies clearly revealed the presence of the shorter His_6_-tagged *A* subunit, corresponding to a ∼65 kDa band and its faster- and slower-migrating forms, respectively corresponding to the mature glycosylated (Papazian et al., 1995; Tiwari-Woodruff et al., 1997) and truncated versions of the protein. The opposite pattern of antibody binding was observed when the experiment was repeated, this time however, with native Kv channel particles immobilized to a His-trap affinity column *via* the His_6_-tagged *A* subunit and probed with anti-His or anti-FLAG antibodies (Figure 2C). Whereas the upper panel clearly reveals the typical bands of the *A* subunit in the elution and other fractions, thus confirming the success of the *A* subunit-based immobilization step, the lower panel involving detection *via* the anti-FLAG antibodies reveals two primary bands corresponding to the *B* subunit, specifically the mature glycosylated (∼90 kDa) and truncated versions of the protein (∼57 kDa). Such dual antibody detection of both the *A* and *B* subunits within the same Kv channel particle was not observed in control experiments in which native membrane protein fractions of cells expressing either the *A* and *B* subunit variants alone were combined prior to the subunit-based pull-down immobilization steps and subsequent detection steps described above (not shown). Taken together, our results indicate the presence of hetero-oligomeric Kv channel particles composed of both short (*A*) and long (*B*) clustering chain variants.

**FIGURE 3:**
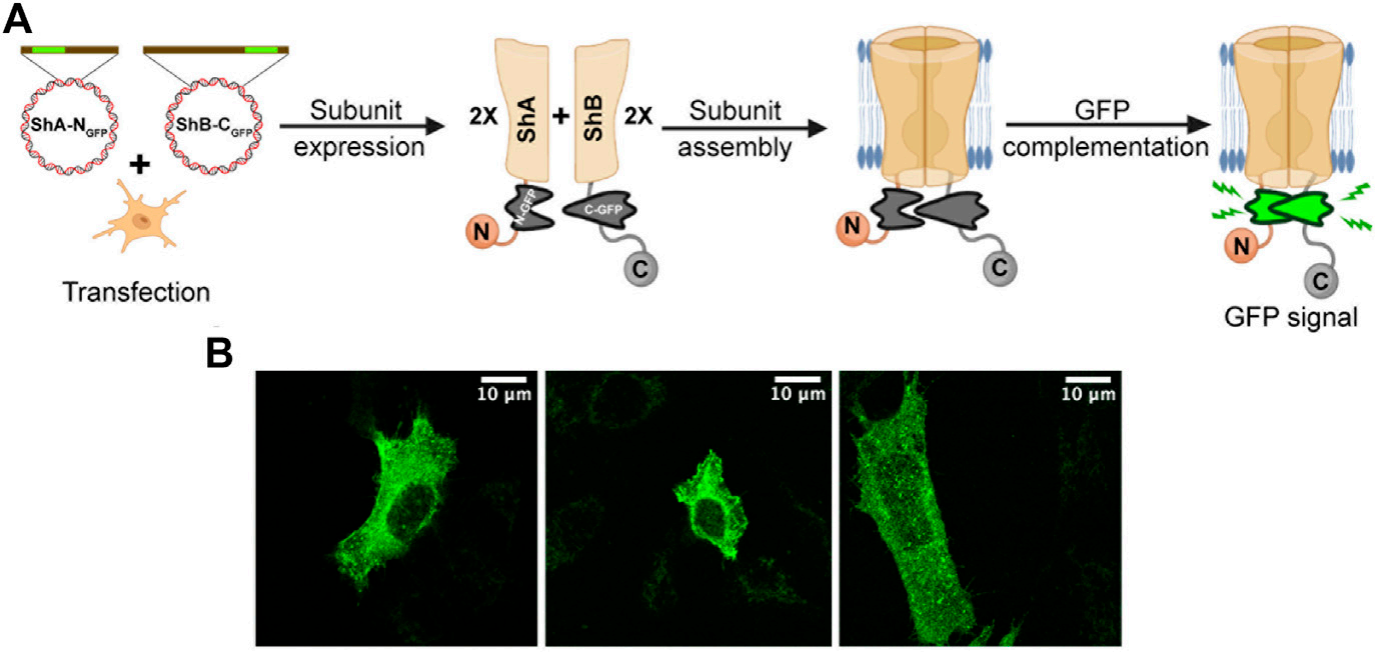
Direct demonstration of hetero-oligomeric Kv channel assembly. **(A)** Schematic representation of the GFP complementation assay used to directly demonstrate hetero-oligomeric assembly of Kv channels composed of both the *A* and *B* subunits (see text). **(B)** Confocal microscopy images of three distinct SH-SY5Y cells transfected with DNA encoding both the *A* and *B* channel variants (at a 1:1 ratio), each fused to a half of GFP (N_GFP_ and C_GFP_, respectively fused to the *A* and *B* subunits). The green fluorescence signal directly indicates intra-molecular assembly of the *A* and *B* subunits to form hetero-oligomeric channels.

Despite the clear-cut nature of the results reported above, they are, non-etheless, based on indirect evidence and, moreover, involved *in vitro* biochemical analysis outside the native cellular context. We thus sought a direct manner to observe Kv channel hetero-oligomerization *in vivo*, *i.e.*, within the cellular membrane context. For this purpose, we used the split GFP bio-complementation assay (Figure 3A) (Kerppola, 2008). Briefly, DNA encoding for either of the two structurally complementary N- and C-terminal halves of GFP (N_GFP_ and C_GFP_) were inserted into DNA encoding either the *A* or *B* subunits at different sites known to encode regions of the proteins that are in close spatial proximity. The insertion sites were chosen based on the low-resolution electron microscopy structure of the native *Shaker K*v channel (Sokolova et al., 2001). DNA coding for both N_GFP_-*Shaker A* and *Shaker B*-C_GFP_ fusion proteins was then introduced into SH-SY5Y cells. Following protein subunit expression, the cells were imaged by high-resolution confocal microscopy, as described in the Materials and Methods section and in Lewin et al. (2020). In this experimental setup, a green fluorescence signal within the imaged cells should appear only if three criteria are met: (1) The two fusion subunit variants are properly expressed; (2) both *A* and *B* channel variants co-assemble to form a hetero-oligomeric Kv channel; and (3) GFP complementation occurs in the hybrid channel particle context. In other words, the appearance of a green fluorescent signal would provide direct indication for hetero-oligomeric Kv channel assembly involving both the *A* and *B* subunits (Figure 3A).

Confocal microscopy images of SH-SY5Y cells transfected to express equal amounts of both subunit variants revealed a strong GFP signal pattern throughout the cell membrane (Figure 3B). No such signal was observed when either the *A* or *B* fusion constructs was expressed alone (not shown). Furthermore, the diffuse green signal observed indicated the presence of many Kv channel molecules distributed throughout the cell membrane, rather than being clustered at specific sites, as do native Kv channels when expressed in the absence of the PSD-95 scaffold protein partner (Lewin et al., 2020). The absence of PSD-95 in this experiment was necessary to rule out the possibility that the green fluorescence signal resulted from inter-molecular interactions among adjacent *A* and *B* subunit-containing channel particles residing within the same clustering site. The images presented in Figure 3B thus provide direct evidence for hetero-oligomeric *Shaker* Kv channel assembly.

Combined, the results of both the *in vitro* biochemical and *in vivo* biophysical analyses indicate that hetero-oligomeric Kv channel particles composed of both the long and short clustering ‘chain’ subunit variants exist, thus complementing electrophysiology recording indirectly pointing to such assembly (McCormack et al., 1990). Finally, although we cannot currently determine the numbers of the different *A* and *B* subunit combinations in the different hetero-oligomers, all hybrid *A/B* species are possible, as described by binomial distribution, in particular considering that both the *A* and *B* variants include an identical T1 assembly domain, as well as a membrane-spanning segment (Kreusch et al., 1998).

### *Shaker* channel hetero-oligomerization regulates cluster Kv channel density

**FIGURE 4:**
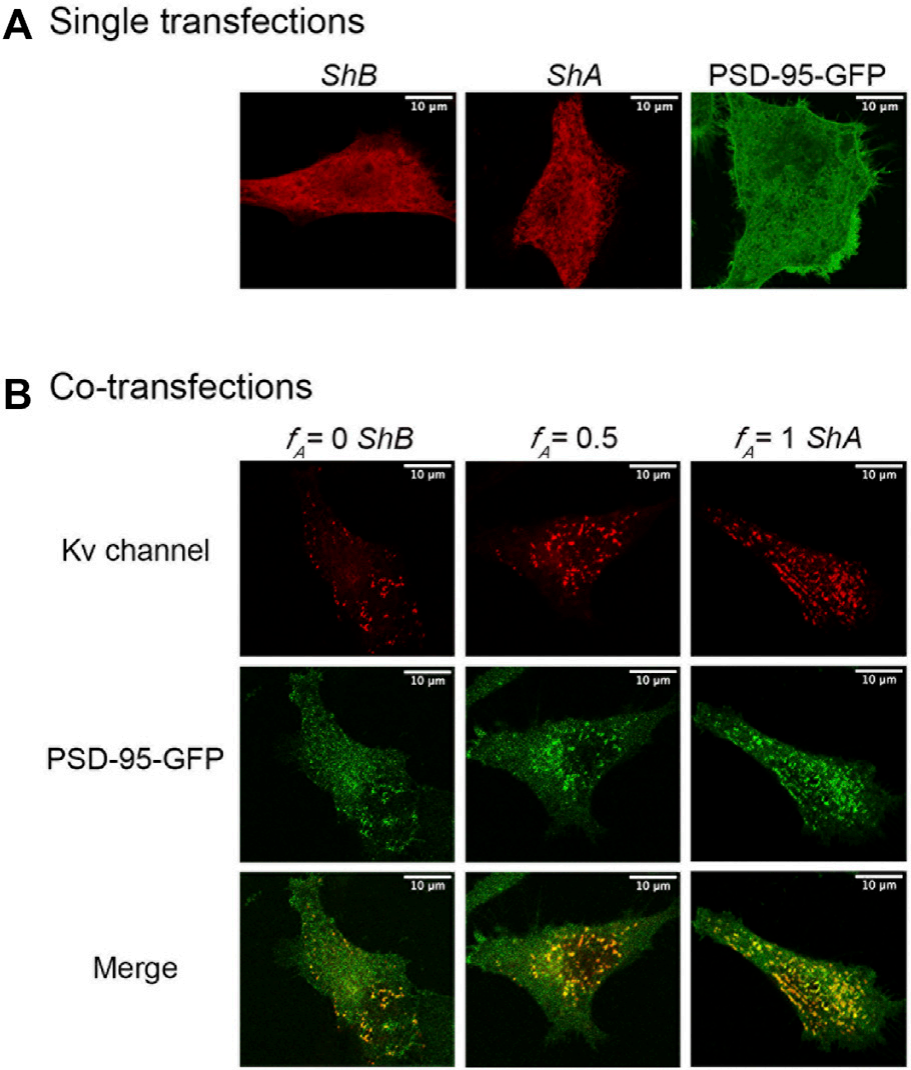
Confocal microscopy imaging of cellular Kv channel clustering. **(A)** High-resolution confocal microscopy images of SH-SY5Y cells expressing either the native *Shaker A* or *B* Kv channel variants alone or with the PSD-95-GFP scaffold protein partner (see Materials and Methods). **(B)** Typical images of cells co-expressing either the *A* or *B* native channel variants or a 1:1 combination thereof and PSD-95-GFP are shown in the left, right and middle columns, respectively. Three images are shown for each cell, with the red channel-associated fluorescent signal shown in the top row, the green PSD-95-associated signal presented in the middle row, and the merged image shown in the bottom row. Typical triplicate images of cells transfected to express different *A*:*B* subunit DNA concentration ratios are shown in Supplementary Figure S2. Acquisition conditions for all samples were identical (see Materials and Methods). Note that under these conditions, it is the membrane surface of the cells that is imaged and so the nucleus is not seen. When the cell is imaged at the equatorial plane cross section, the nucleus is visible and exhibits rim fluorescence, reflecting Kv channel accumulation at the membrane of the nucleus (not shown). Scale bars in panels **a** and **b** and in all images presented in Supplementary Figure S2 correspond to 10 μm.

Previous results obtained using the native (*A* and *B*) and artificial chain length clustering variants indicated that Kv channel C-terminal ‘chain’ length modulates clustering site Kv channel density in a manner determined by the affinity of the 'chain' to its partner PSD-95 scaffold protein (Lewin et al., 2019; Lewin et al., 2020). Considering the series of Kv channel particles exhibiting increasing numbers of the short, high-affinity *A* clustering subunit presented in Figure 1C, it can be assumed that heterologous subunit assembly could serve as a means to regulate Kv channel clustering. To test this assertion, we combined our channel clustering imaging setup with a powerful clustering metrics methodology (see Materials and Methods) (Lewin et al., 2020). Briefly, SH-SY5Y cells were co-transfected to express increasing molar concentration ratios of FLAG-tagged *A* (high-affinity, short) and *B* (low-affinity, long) channel variants, either alone or together with PSD-95-GFP. An identical overall subunit concentration was used in each case Supplementary Figure S1. At the same time, systematically higher *A:B* subunit transfection ratios would be expected to increase the propensities of channel combinations comprising more *A* subunits, as dictated by binomial distribution (Figure 1C). This is particularly true considering that the *A* and *B* subunit share identical T1 assembly and core transmembrane domains (Kreusch et al., 1998), and thus no preference for the subunit pairs assembled (i.e., *AA, AB,* or *BB*) would be expected. Support for this latter argument comes from a seminal study on the subunit stoichiometry of tetrameric Kv channels assembled from wild type and mutant subunits (MacKinnon, 1991). We thus used the molar transfection fraction of the high-affinity *Shaker A* subunit (*f*_A_ = [A]/([A]+[B])) as the axis upon which hetero-oligomeric Kv channel affinity for PSD-95 was tuned (achieved by modulating the number of high-affinity *A* subunits within hybrid oligomers) and tested for effects on cell-level and site-level clustering. Following fixation, permeabilization and immunostaining of (cytoplasmic-oriented) tagged-channel epitopes, the transformed cells were imaged by focusing directly on the ‘cover slip’-attached basal membrane plane. As elaborated in the Materials and Methods and in more detail in our previous study (Lewin et al., 2020), it is the membrane surface that is imaged under these acquisition conditions. Typical confocal microscopy images of the *A* and *B* channel variants and of channels in which these variants are found at a 1:1 concentration ratio (i.e., *f*_A_ = 0.5) are provided in Figure 4 (for images obtained for all *f*_A_ transfection fractions examined, see Supplementary Figure S2). Control experiments involving cells transfected to express either of the *Shaker A* or *B* channels alone, or just PSD-95-GFP, showed homogeneous diffuse labeling, reflected by the *Shaker* channel-associated red fluorescent pattern or the PSD-95-associated green fluorescent signal (Figure 4A), as previously demonstrated (Lewin et al., 2020). However, co-expression of PSD-95-GFP and either of the *Shaker* channel variants or their 1:1 molar ratio (Figure 4B) (or all other transfection ratios (Supplementary Figure S2)) resulted in a speckled pattern. In agreement with previous analyses (Lewin et al., 2020), the membrane-associated yellow coloring of the merged images reflects protein co-localization and clustering, with the bright yellow spots in each image representing Kv channel clustering sites having different shapes, area sizes and densities. Inspecting the data, however, revealed apparent differences in co-localization and clustering patterns among the different transfection treatments.

**FIGURE 5:**
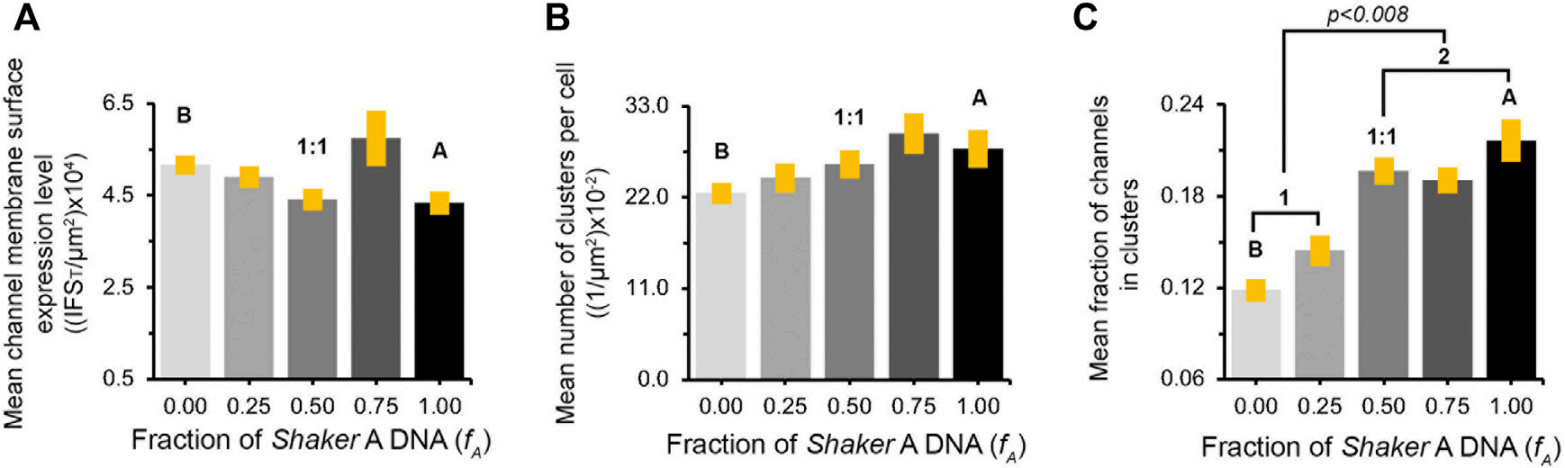
Similar channel cell surface expression patterns appear at all *A*:*B* subunit transfection ratios. **(A)** Comparison of mean channel membrane surface expression levels in cells transfected to express different concentration ratios of the *A* and *B* Kv channel subunit variants and the PSD-95 scaffold protein. Channel membrane surface expression levels are reflected in the integrated channel-associated red fluorescence signal within the cell (IFS_T_), normalized to cell area. **(B)** Comparison of the mean number of clusters per cell (normalized to cell area) in cells transfected to express the different *A*:*B* subunit variant transfection ratios and the PSD-95 scaffold protein. The values in both analyses presented in **(A)** and **(B)** were found to be similar, based on multi-variant ANOVA for multiple comparisons. In each case, the *p* values for all comparisons were higher than 0.03. **(C)** Comparison of the mean fraction of the *A* or *B* Kv channel variants or combinations thereof (as modulated by different *A*:*B* transfection ratios), expressed at clustering sites. The values were obtained by dividing the overall channel-associated red signal within cell clustering sites (IFSc) to IFS_T_. The labels *1* and *2* refer to the two distinct, low and high, IFSc/IFS_T_ groups. See Materials and Methods and the text for details on how these cell-based clustering attributes were calculated.

**FIGURE 6:**
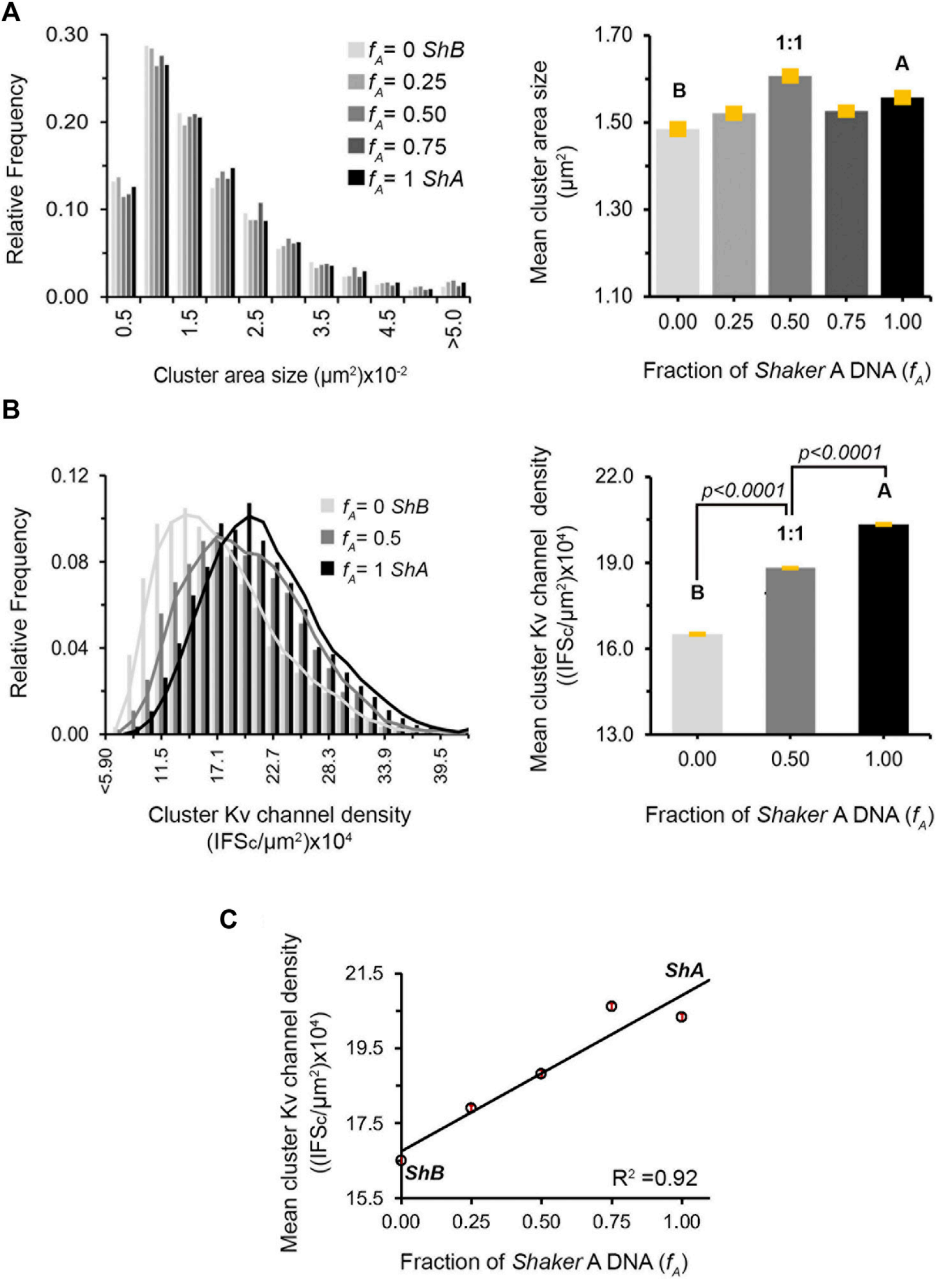
Regulation of clustering site Kv channel density by heterologous subunit assembly. **(A)** Cluster Kv channel area size distributions of cells transfected with different *A*:*B* subunit DNA concentration ratios, as evaluated by clustering analysis and supported by PSD-95. Distributions in steps of 0.5 (x10^−2^) μm^2^ are presented (*n* = 3000–5,000 clusters). Comparison of the mean values of cluster ion channel area size for the different *A*:*B* subunit ratio treatments is given in the right panel. Values were all found to be similar, based on ANOVA (the *p* values for all comparisons are higher than 0.1) **(B)** Cluster Kv channel density distributions of cells transfected with DNA encoding for the *Shaker A* or *B* channels alone or at the 1:1 *A*:*B* subunit DNA transfection ratio, as evaluated by cluster signal intensity and supported by PSD-95, are shown in the left panel. Distributions in steps of 2.8 (x10^4^) IFSc/μm^2^ are presented (*n* = 3,000–5,000 clusters). Cluster Kv channel density distributions for all *A*:*B* subunit DNA transfection ratios are presented in Supplementary Figure S3. Comparison of the mean values of cluster ion channel density for the different *A*, *B* subunit transfections or their 1:1 combination are shown in the right panel. All differences were found to be statistically significant, based on ANOVA for multiple comparisons (the *p* values for all three comparisons are smaller than 0.0001). **(C)** Dependence of the mean value of PSD-95-mediated cluster Kv channel density on *A*:*B* subunit DNA transfection ratio. Differences in cluster ion channel density were all found (but for the 0.75 and 1 value pair) to be statistically significant, based on ANOVA (*n* = ∼3,000–5,000; *p* < 0.0001).

To quantify the effect(s) of the different short *A* to long *B* 'chain' molar concentration ratios on the PSD-95-mediated Kv expression and clustering seen in Figure 4 and Supplementary Figure S2, multiple images (*n* = ∼23) of each population were collected and subjected to clustering metrics analysis to evaluate and compare all clustering attributes, including the mean channel membrane surface expression level, mean number of clusters per cell, mean fraction of channels in clusters, mean cluster site area size and mean clustering site Kv channel density, as described in Materials and Methods and in Lewin et al. (2020). The results of such analysis are presented in Figure 5 for cell-level clustering attributes and in Figure 6 for 'clustering site'-level attributes, as well as being further summarized in Table 1.

As can be seen in Figure 5A, all the different variant molar transfection ratios that gave rise to systematically higher *f*_A_ values exhibited similar mean channel membrane expression levels, as calculated by the total red channel-associated cell fluorescence signal normalized to cell area (Table 1). Secondly, for all *f*_A_ values, no differences were observed for the mean number of clusters per cell either, again normalized to cell area (Figure 5B; Table 1). Third, as can be seen in Figure 5C and reported in Table 1, the mean fraction of channels within clusters (calculated by the fraction of red channel-associated signal residing in clustering sites) appeared to differ as a function of the *A*:*B* subunit transfection ratio. Although differences in this value cannot be clearly resolved at the single *f*_A_ value level, two statistically distinct groups of low- and high-fraction values (designated 1 and 2 in Figure 5C) were clearly revealed in the analysis (*p* = 0.008). In considering clustering site-level attributes, statistical analysis of 3,000–5,000 clusters of each *A*:*B* transfection ratio used, obtained from ∼23 different cell images, revealed similar distributions of cluster area sizes, irrespective of the *A*:*B* molar concentration transfection ratio used (Figure 6A, left panel). This is further reflected in the similar mean cluster area size values detected (Figure 6B, right panel; *p* > 0.1 for all but one pairwise comparisons). At the same time, the distributions of cluster ion channel density (reflected in cluster signal intensity; see Materials and Methods) for the different ratios used were computed and are presented in Supplementary Figure S3, with several being further compared in the left panel of Figure 6B. As can be seen, the distributions of cluster ion channel density of all transfection ratios appeared non-normal (slightly skewed to the right) and presented different cluster Kv channel density ranges (Supplementary Figure S3), with that of the short ‘chain’ homo-tetrameric *A* and the long ‘chain’ homo-tetrameric *B* variants being displaced towards highest and lowest channel densities, respectively. This is also clearly demonstrated in Figure 6B (left panel), where the distributions of cluster ion channel density of cells transfected to express only the *A* (*f*_A_ = 1) or *B* (*f*_A_ = 0) ‘chains’ or a 1:1 molar concentration ratio thereof (*f*_A_ = 0.5) are directly compared. The distribution of the 1:1 molar ratio treatment was between both the extreme values seen with each homogenous channel (see figure legend for statistical support of this assertion). This is further reflected by noting that the mean values of cluster ion channel density for all the different *A*:*B* molar transfection ratios (except those of *f*_A_ = 1 and the *f*_A_ 0.75 pair), were all found to be statistically distinct (*n* = 3,000–5,000; *p* < 0.0001; see Materials and Methods) (Figure 6B, right panel). Last, yet importantly, a linear dependence between the mean clustering site Kv channel density and the fraction of *Shaker A* subunit was revealed (Figure 6C; *R* ^2^ = 0.92), such that the higher the propensity to incorporate more copies of the short 'chain', high-affinity *A* subunit variant into the hybrid tetrameric Kv channel species, the higher was the clustering site Kv channel density observed. Taken together, the results described here indicate that hetero-oligomeric subunit assembly indeed offers a means to regulate the process of Kv channel clustering and, in particular, the density of potassium channels and the underlying potassium currents that pass at their sites of expression.

## Discussion

In the current manuscript, our primary concern was the manner in which potassium current density through Kv channels is regulated, given how such regulation is of profound importance for electrical signaling and information coding. Such regulation can be brought about by modulating the copy number of Kv channel molecules at their sites of expression, as achieved by PSD-95-mediated channel clustering. We have previously shown that alternative splicing of the *Shaker* Kv channel gene gives rise to homo-tetrameric *A* and *B* channel molecules and thus serves to regulate cluster Kv channel density (Lewin, et al., 2020). Moreover, it was found that the short chain *A* variant presented higher cluster Kv channel density, as compared to the long chain *B* variant. Bearing in mind that both clustering variants are expressed in the same fly tissues and at similar developmental stages, the possibility of heterologous subunit assembly as another dimension for cluster Kv channel density regulation is intriguing. We hypothesized that the series of hybrid Kv channel molecules comprising increasing numbers of the short high-affinity *A* subunit would be expected to present a systematic increase in affinity to the PSD-95 partner protein, giving rise to progressively higher cluster Kv channel densities (Figure 1C).

The results presented in this manuscript validate this hypothesis and point to Kv channel hetero-oligomerization as a means to regulate K^+^ current densities at Kv channel clustering sites. First, we directly demonstrated that the formation of hetero-multimeric channel molecules composed of both *A* and *B* clustering subunits is possible. Using 'affinity column'-based pull-down analysis under native conditions that support proper membrane protein assembly and folding, we showed that channel particles immobilized to the column *via* one subunit can be detected using antibodies against the second subunit. Such detection was demonstrated in both directions (Figure 2). Second, a split GFP bio-complementation assay performed under native cellular conditions demonstrated that both 'half-GFP′-fused *A* and *B* subunits can be found in the same channel molecule, where they intra-molecularly interact to give rise to the typical diffusive pattern of GFP signal characteristic of non-clustered Kv channels (Figure 3; Lewin et al., 2020). Thus, both our *in vitro* and *in vivo* analyses suggest that hybrid Kv channel molecules exist.

Despite the results described above, it still remained to be shown that controlling the number of high- or low-affinity subunits serves to regulate channel clustering. For this, we studied the effects of transfecting increasingly higher *A*:*B* molar concentration ratios into the SH-SY5Y model cell system on Kv channel clustering. The molar transfection ratio axis (or its corresponding *f*_*A*_ value) serves to tune hetero-oligomeric Kv channel affinity by modulating the number of high-affinity *A* subunits within binomially distributed heteromeric channel combinations. While such DNA-level manipulation is far upstream of what is found at the protein-level and in terms of the clustering process being evaluated, we took careful care to ensure that all clustering-affecting manipulations were subsequently measured under similar acquisition conditions, thereby minimizing expression biases across transfections (see Materials and Methods), as confirmed experimentally (see below). Interestingly, at all *A*:*B* DNA transfection ratios employed, similar values were obtained for channel cell surface expression (Figure 5A). This observation might seem trivial, as identical overall subunit amounts were used in each transfection, however, it implies that no expression bias existed for any variant transfection ratios when progressing from the DNA to the protein level. Likewise, similar channel membrane expression levels were also found for a series of Kv channel deletion mutants presenting increasingly shortened C terminal ‘chains’, evaluated using the same experimental design as described here (Lewin et al., 2020). Thus, changes in channel variant affinity to PSD-95, brought about by changes in 'chain' length or by changes in the number of high-affinity *A* subunits within the heteromeric channel, do not affect channel expression level. This observation is further strengthened by our finding that at all transfection ratios examined, similar mean numbers of clusters per cell were obtained (Figure 5B). Although similar overall channel membrane expression levels were observed for all transfection ratios, the manner in which the red channel-associated signal was distributed between clustering sites or appeared freely diffused in the membrane differed. Specifically, higher *A*:*B* transfection ratios gave rise to an increase in the fraction of channels residing within clusters (Figure 5C). This cell-level observation implies that clustering is affected by increasing the number of high-affinity *A* subunits within the hetero-oligomeric Kv channel.

Examination of the clustering site-level statistics revealed that although the mean value of cluster area sizes showed no differences among the different transfection ratios used (Figure 6A), the mean cluster Kv channel density value did (Figure 6B). A linear empirical correlation between the two quantities was noted, with higher *A*:*B* DNA transfection ratios (i.e., higher *f*_A_ values) giving rise to progressively higher cluster Kv channel densities (Figure 6C). Thus, increasing the weighted affinity of all heterologous Kv channel subunit combinations presenting higher numbers of the *A* subunit, achieved by increasing the *A*:*B* transfection ratio, gave rise to increased densities of Kv channel molecules within clustering sites, pointing to an increase in the copy number of channel molecules. This observation is further supported by the recent demonstration of a monotonic linear dependence between cluster Kv channel density of the homo-tetrameric *B* channel and the affinity of the channel-PSD interaction pair, as modulated by systematic shortening of the *B* channel C-terminal region (Lewin et al., 2020). In this case, however, artificially shortening the chain too much led to a decrease in cluster Kv channel density due to steric hindrance considerations, related to the inability of the multiple PDZ domains of PSD-95 to bind adjacent channel molecules having too short tails. In nature, such extremely short chains do not exist, such that no bell-shaped behavior is observed upon heterologous channel assembly.

Taken together, our results reveal that heterologous subunit assembly offers a means to regulate cluster Kv channel density. Specifically, spatial and temporal tuning the expression levels of the short (*A*) or long (*B*) or both C-terminal native ‘chain’ variants (Mottes and Iverson, 1995; Rogero, et al., 1997) would thus allow channel variability upon hetero-oligomeric subunit assembly (McCormack., et al., 1990) and give rise to channels with distinct affinities to PSD-95, in turn, leading to distinct PSD-95-mediated Kv channel densities. Such modulation, as demonstrated here, would lead to changes in ionic current density at the sites of homo- or hetero-oligomeric channel localization, such as at the post-synaptic density. This could subsequently lead to changes in action potential transmission and frequency, and synaptic growth and plasticity (Århem, et al., 2006; Zeberg, et al., 2010; Zeberg, et al., 2015).

The results described here regarding splicing-based regulation of *Shaker* Kv channel clustering *via* subunit hetero-multimerization may also be relevant to mammalian Kv channels. Although mammalian Kv channel subunits do not result from alternative splicing of the C-terminal tail-encoding message, different Kv channel subunit paralogues exist which exhibit differences in the lengths of the C-terminal 'chain' that contains terminal PDZ-binding motifs that recruit PSD-95 scaffold proteins (Magidovich, et al., 2006). Furthermore, phylogenetic inference analyses of the Kv channel family revealed how the intrinsic disorder of the channel C-terminal tail has co-evolved with the appearance of the terminal PDZ sequence motif, thus arguing for conservation of the ' ball and chain'-like mechanism for the PSD-95 binding underlying mammalian Kv channel clustering. Moreover, heterologous assembly of such paralogues has been reported (Clarke and Gulbis, 2012; Trimmer, 2015). Whether or not this is indeed the case will require further analysis.

In summary, the *Shaker* Kv channel model protein discussed here, with its two native clustering subunit variants, provides a clear example of how molecular distinctions reflected in the different interactions of the C-terminal ‘chain’ variants and combinations thereof with PSD-95 translate into functional differences in the context of cellular channel clustering. Understanding the linkage between the two levels of organization and its physiological implications, as exemplified here, is only possible when knowledge of the molecular mechanism underlying Kv channel-scaffold interaction is available, thus serving to bridge the molecular-cellular gap in our understanding of Kv channel clustering.

## Data Availability

The original contributions presented in the study are included in the article/Supplementary Material, further inquiries can be directed to the corresponding author.

## Supplementary material

SUPPLEMENTARY FIGURE S1 Plasmid maps and nomenclature of all DNA constructs used in the different analyses performed in the current study.

SUPPLEMENTARY FIGURE S2 High-resolution confocal microscopy images of SH-SY5Y cells transfected with the DNA of the PSD-95-GFP scaffold protein along with different DNA transfection ratios of the *A* and *B* subunit variants to produce homo- and hetero-tetrameric Kv channel combinations. Typical images of cells co-transfected with increasing *A*:*B* subunit transfection ratios and with PSD-95-GFP are shown from the left to right columns. Three images are shown for each cell, with the red channel-associated fluorescent signal shown in the upper row, the green PSD-95-associated signal presented in the middle row, and the merged shown in the lower row. Acquisition conditions for all samples were identical (see Materials and Methods). Scale bars in all images correspond to 10 μm.

SUPPLEMENTARY FIGURE S3 Cluster Kv channel density distributions of cells transfected with different *A*:*B* subunit concentration ratios, as evaluated by cluster signal intensity and supported by PSD-95. Distributions in steps of 1.4 (x10^4^) IFSc/μm^2^ are presented (*n*=3,000–5,000 clusters). See Figure 6 legend and Materials and Methods for statistical validation of the observed differences in cluster Kv channel density among the different transfection ratio treatments.
