# Supplementary material for: Regulating Shaker Kv channel clustering by hetero-oligomerization
Source: Front Mol Biosci. 2023 Jan 9;9:1050942. doi: 10.3389/fmolb.2022.1050942 (PMC9868669; doi:10.3389/fmolb.2022.1050942)
Supplement: Supplementary file 1 [file DataSheet1.PDF]

Supplementary Information Figure 1: Plasmids used in the current study

#	Plasmid[a]	Description	Analysis purpose
1	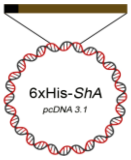	His-tagged *Shaker A* channel	Pull-down assay
2	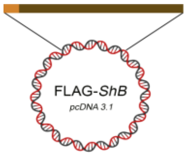	FLAG-tagged *Shaker B* channel	Pull-down assay
3	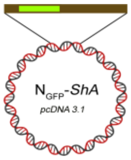	N_{GFP}-*Shaker A* fusion construct	Split GFP bio-complementation assay
4	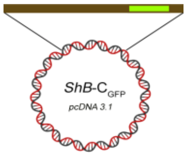	C_{GFP}-*Shaker B* fusion construct	Split GFP bio-complementation assay
5	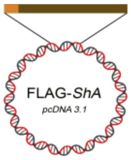	FLAG-tagged *Shaker A* channel	High-resolution confocal microscopy imaging
6	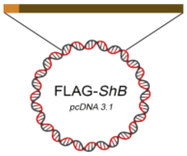	FLAG-tagged *Shaker B* channel	High-resolution confocal microscopy imaging of channel clustering
7	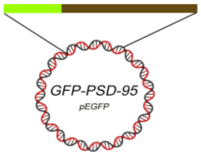	GFP-PSD-95 fusion construct	High-resolution confocal microscopy imaging of channel clustering

[a]All Kv channel containing plasmids are based on the pcDNA vector. Insert *Shaker* Kv channel subunits are indicated in brown color, GFP in green, His tag in black and FLAG tag in orange.
